# Potential drawbacks of frequent asymptomatic coronavirus disease 2019 (COVID-19) testing

**DOI:** 10.1017/ice.2020.1305

**Published:** 2020-10-29

**Authors:** Giuseppe Lippi, Brandon M. Henry, Fabian Sanchis-Gomar

**Affiliations:** 1 Section of Clinical Biochemistry, University of Verona, Verona, Italy; 2 Cardiac Intensive Care Unit, The Heart Institute, Cincinnati Children’s Hospital Medical Center, Ohio, United States; 3 Department of Physiology, Faculty of Medicine, University of Valencia and INCLIVA Biomedical Research Institute, Valencia, Spain

*To the Editor—*Although significant emphasis has been given to widespread community screening for identifying severe acute respiratory syndrome coronavirus 2 (SARS-CoV-2) infection, also in asymptomatic people, Bai et al^1^ recently concluded that this strategy would not add clinically useful information, nor would have significant impact on current infection control management. The theoretical benefits of population coronavirus disease 2019 (COVID-19) screening include achieving more information for forecasting pandemic evolution, optimizing and quickening the establishment of preventive and containment strategies, and accurately and rapidly assessing the efficiency of implemented measures.^2^ On the other hand, there are also some potential drawbacks that may emerge from mass testing of asymptomatic patients.

Currently, reagent availability is the most limiting aspect for implementation of large-scale testing. A recent survey of the American Association of Clinical Chemistry (AACC) has revealed that >50% of worldwide clinical laboratories were still facing dramatic shortages of test kits and reagents at the end of September 2020, with >70% of respondents emphasizing substantial challenges to increase their testing capacity.^3^ The gold standard for diagnosing COVID-19 is identification of viral RNA in upper respiratory tract samples, collected and tested by skilled and trained healthcare personnel. Thus, staff shortages will further complicate the possibility of amplifying the actual testing volume, which remains now insufficient for even testing all suspect and symptomatic subjects in many worldwide regions. Therefore, specimen collection and reagent shortages have inhibited rapid increases in testing capacity, representing a bottleneck and critical limitation in intensifying SARS-CoV-2 diagnostic testing.

The widespread identification of several hundred thousand, or even millions, of asymptomatic people, representing now the vast majority of SARS-CoV-2–positive cases in certain regions, is a second important aspect. It is now undeniable that the infectivity of asymptomatic COVID-19 patients is weaker and progressively declines over time.^4^ Therefore, mandatory isolation of a massive number of people, who are less likely to substantially contribute to transmitting the virus even when positive (the secondary attack rate of asymptomatic people has been reported at around 3%),^5^ especially when all the recommended preventive measure are adopted (ie, social distancing, hand hygiene, use of face masks, avoid singing or shouting), will likely bring forth further negative impacts on society, economy, and even healthcare, whereby isolation of many asymptomatic physicians and other healthcare providers would impair the possibility to deliver standard care.^6^ There are also important psychological consequences from the quarantining of asymptomatic COVID-19 subjects, who may develop a wide array of disturbances such as psychological distress and declining daily functioning.^7^ Moreover, questions arise as to whether “high-risk” contacts of asymptomatic individuals (ie, household family members, etc), would also require quarantine, further complicating a mass testing strategy.

Finally, diagnosing asymptomatic COVID-19 people, who are unlikely to develop medium- or long-term consequences from this infection, could expose these people to further invasive and potentially harmful testing (eg, radiation) and even unnecessary treatments, which may be associated with undesirable side effects that could be worse than the disease itself.^8,9^ Rates of false-negative swab tests between 2% and 33% in repeat sample testing have been reported, while rates of false positives have been estimated between 0.8% and 4.0%, probably due to technical problems such as contamination during sampling, contamination of amplicons or reagents, and cross reactions with other viruses or genetic material.^10^ Assuming a false-positive rate of only 1%, for every 1 million tests run per day, 10,000 false-positive results would occur. This, combined with the detection of tens of thousands of asymptomatic individuals daily at lower risk of transmitting the virus in the presence of appropriate precautions, would likely overwhelm our ability to effectively contact trace and rapidly contain the highest-risk clusters.

No evidence-based data for universal screening of asymptomatic COVID-19 patients has been reported so far. Although we agree that strengthened molecular and/or antigenic COVID-19 testing of symptomatic subjects and their contacts represents a mainstay for pandemics containment, additional evidence and development of new public health strategies to handle the results of such testing would be needed before massive testing of asymptomatic COVID-19 individuals could be recommended (Table 1). Importantly, testing nonsymptomatic individuals may also cause false sense of security. To date, universal precautions such as hand and respiratory hygiene, self-quarantine when symptomatic or possible contact, social distancing, and use of masks are the best methods to mitigate COVID-19.

**Table 1.** Potential Drawbacks of Widespread Asymptomatic COVID-19 Testing

• Shortage of supplies and human resources for testing symptomatic patients and for contact tracing
• Unnecessary isolation of subjects with non-progressive disease and lower infectivity • Adverse economic, societal and healthcare consequences • Physiological distress
• Risk of overdiagnosis and overtreatment
